# In vitro and in vivo evaluation of selected ^68^Ga-siderophores for infection imaging^☆^

**DOI:** 10.1016/j.nucmedbio.2011.09.012

**Published:** 2012-04

**Authors:** Milos Petrik, Hubertus Haas, Markus Schrettl, Anna Helbok, Michael Blatzer, Clemens Decristoforo

**Affiliations:** a Clinical Department of Nuclear Medicine, Innsbruck Medical University, Innsbruck, Austria; b Division of Molecular Biology/Biocenter, Innsbruck Medical University, Innsbruck, Austria

**Keywords:** Siderophores, *Aspergillus fumigatus*, ^68^Ga, PET imaging, Invasive aspergillosis

## Abstract

**Introduction:**

Siderophores are low-molecular-mass iron chelators serving as iron transporters for almost all bacteria, fungi and some plants. Iron is an essential element for majority of organisms and plays an important role in virulence of pathogenic organisms. ^68^Ga is a positron emitter with complexing properties comparable to those of Fe(III) and readily available from a generator. Initial studies with ^68^Ga-triacetylfusarinine C (TAFC) showed excellent targeting properties in a rat infection model. We report here on the in vitro and in vivo evaluation of other siderophores radiolabelled with ^68^Ga as potential radiopharmaceuticals for infection imaging.

**Methods:**

^68^Ga labelling was performed using acetate buffer. Stability, log *P* and protein binding values were determined. In vitro uptake was tested using iron-deficient and iron-sufficient *Aspergillus fumigatus* (*A.f.*) cultures. Biodistribution of ^68^Ga-siderophores was studied in Balb/c mice.

**Results:**

Significant differences among studied siderophores were observed in labelling efficiency, stability and protein binding. Uptake in *A.f.* cultures was highly dependent on iron load and type of the siderophore. In mice, ^68^Ga-TAFC and ^68^Ga-ferrioxamine E (FOXE) showed rapid renal excretion and low blood values even at a short period after injection; in contrast, ^68^Ga-ferricrocin and ^68^Ga-ferrichrome revealed high retention in blood and ^68^Ga-fusarinine C showed very high kidney retention.

**Conclusions:**

Some of the studied siderophores bind ^68^Ga with high affinity and stability, especially ^68^Ga-TAFC and ^68^Ga-FOXE. Low values of protein binding, high and specific uptake in *A.f.*, and excellent in vivo biodistribution make them favourable agents for *Aspergillus* infection imaging.

## 1 Introduction

Siderophores are low-molecular-weight (500–1500 Da), iron-chelating molecules produced by nearly all bacteria, fungi and some plants [1]. Since 1970, a large number of siderophores have been characterized. The majority possess hydroxamate, catecholate or α-hydroxycarboxylate functional groups and form six coordination complexes with extremely high affinity (binding constant of >10^30^) and selectivity for ferric ions [1]. Their biosynthesis is regulated by the iron levels of the environment where the organism is located, and they serve to deliver iron into the microbial cells [2].

Iron is an essential nutrient for almost all organisms. For prime producers, such as bacteria, fungi and plants, iron bioavailability is limited by the inherently low solubility of ferric ions. Under aerobic conditions, iron exists mainly in the form of Fe(III), as hydroxide and oxyhydroxide colloid particles that have a solubility below 10^−9^ M at neutral pH [3]. This is far below the level of demand for the iron supply of living cells. Therefore, iron-dependent microorganisms have evolved different strategies to solve the bioavailability problem. These strategies usually involve biosynthesis of siderophores. Extracellular siderophores serve microorganisms to acquire iron from the environment, while intracellular siderophores have been proposed to play a role in iron storage and have been recognized as asexual spore germination factors of several microorganisms (*Neurospora crassa*, *Penicillium chrysogenum*, *Aspergillus nidulans*, etc.) [4].

After synthesis and excretion of an iron-free siderophore (desferri-siderophore) followed by chelation of iron, the siderophore–iron complex (ferri-siderophore) is taken up into the cell. Highly specific iron uptake systems [2] recognize the specific siderophore as well as its chirality. They transport the ferric complexes into the cell in an active and energy-dependent way. The ferric ions once collected here are then handed over to the intracellular transport and storage components [5].

In recent years, it has become clear that iron acquisition is also one of the important factors of virulence of pathogenic microorganisms [6]. Schrettl et al. [7] demonstrated that siderophores play a fundamental role as a virulence determinant of *Aspergillus fumigatus (A.f.)*. *A.f.* is one of the most common airborne fungi, and humans constantly inhale numerous conidia of this fungus. Usually, these are eliminated in the immunocompetent host by innate immune mechanisms. However, for the immunosuppressed patients, invasive aspergillosis (IA) mainly caused by *A.f.* represents life-threatening and often fatal infection. The prevalence of IA has increased significantly during the past decades, currently being the most common mold infection worldwide [8,9]. Early diagnosis is critical to a favourable outcome of IA, but is difficult to achieve with currently available diagnostic methods, which lack specificity and/or sensitivity.

*A.f.* produces four structurally different hydroxamate peptide siderophores [7,10]: it excretes fusarinine C (FUS) and triacetylfusarinine C (TAFC) to acquire extracellular iron and employs ferricrocin (FC) and hydroxyferricrocin for hyphal and conidial iron storage, respectively [7,11]. The *A.f.* genome encodes seven putative siderophore transporters [12], five of which are up-regulated during iron starvation conditions [13]. As *A.f.* excretes only two siderophore types, FUS and TAFC, these data indicate either high redundancy of siderophore uptake or additional uptake of structurally different siderophores. In this regard, it is interesting to note that several fungal species are able to utilize siderophores produced by other fungi, termed xenosiderophore, e.g., *Saccharomyces cerevisiae, Candida albicans* and *Aspergillus nidulans* [10,14–16].

^68^Ga is a positron emitter that has recently gained great interest for molecular imaging applications using positron emission tomography (PET) [17]. It is readily available from a radionuclide generator, has a suitable short half-life of 68 min and comparable chemistry to Fe(III). In a proof-of-principle study, we recently showed that a ^68^Ga-labelled siderophore (TAFC) can detect *A.f.* infections in a rat animal model using PET imaging [18]. Consequently, we characterized in this study the in vitro and in vivo uptake of endogenous and selected xenosiderophores and evaluate the potential of these compounds as radiopharmaceuticals for PET imaging of IA.

## 2 Materials and methods

### 2.1 Chemicals

All commercially available reagents were of analytical grade and used without further purification. Desferri-siderophores were obtained from Genaxxon Bioscience (Ulm, Germany). ^68^Ga was gained from a ^68^Ge/^68^Ga generator (IGG; Eckert & Ziegler, Berlin, Germany).

### 2.2 Fungal strains and preparation of *A.f.* cultures

Fungal strains used for in vitro studies were *A.f.* wild-type ATCC46645 (American Type Culture Collection) cultured at 37°C in *Aspergillus* minimal medium containing 1% glucose as the carbon source, 20 mM glutamine as the nitrogen source, salts and trace elements, as described previously [19]. Iron-sufficient media contained 30 mM FeSO_4_. For preparation of iron-deficient media, iron addition was omitted. Iron-deficient conditions were verified by detection of extracellular siderophore production, which is suppressed by iron.

### 2.3 Radiolabelling

^68^Ga was eluted from a ^68^Ge/^68^Ga generator using 0.1N HCl (Fluka, Buchs, Switzerland). Varying amounts (10–40 μg) of desferri-siderophores dissolved in water (1 μg/μl) were mixed with 30–80 μl of sodium acetate (155 mg/ml in water) and 300 μl of generator eluate (10–150 MBq of ^68^GaCl_3_). Reaction mixtures (pH 3–4) were incubated at varying temperatures (RT–80°C) for less than 30 min. After the reaction, 100 μl of sodium acetate was added to increase the pH to 6–7. Radiochemical purity (RCP) of labelled siderophores was analyzed on reverse-phase high-performance liquid chromatography (RP-HPLC) or using instant thin-layer chromatography on silica gel impregnated glass fibres (ITLC-SG).

### 2.4 HPLC and TLC

For determination of radiochemical purity of radiolabelled siderophores, a RP-HPLC gradient method was used, as described previously [18]. ITLC-SG (Pall Corporation, East Hills, NY, USA) using 0.1 M sodium citrate (pH=5) as a mobile phase was used for rapid estimation of the product quality. The retention factor (Rf) of labelled siderophores was 0–0.2 and Rf of free ^68^Ga was 0.8-1.

### 2.5 In vitro characterization of selected siderophores

#### 2.5.1 Log *P*

^68^Ga-labelled siderophore in 0.5 ml phosphate buffered saline was added to 0.5 ml octanol in an Eppendorf tube. The tube was vigorously vortexed over a period of 15 min. An aliquot of both the aqueous and the octanol layers was collected and counted in a γ-counter (WIZARD^2^; PerkinElmer, Waltham, MA, USA). The partition coefficient values were then calculated from obtained data (mean of *n*=6).

#### 2.5.2 Protein binding

For the protein binding assessment, ^68^Ga-labelled siderophores were incubated in fresh human serum at 37°C and analyzed up to 120 min by size-exclusion chromatography (MicroSpin G-50 Columns; Sephadex G-50; GE Healthcare, Buckinghamshire, UK). Protein binding of ^68^Ga-siderophores was determined by measuring the activity distributed between the column and eluate using a γ-counter.

#### 2.5.3 Stability

The stability of prepared ^68^Ga-siderophores was tested by incubation of the reaction mixture in fresh human serum, in 6 mM solution of diethylenetriaminepentaacetic acid (DTPA), as well as in a 0.1 M FeCl_3_ solution at 37°C up to 120 min. After incubation, human serum samples were precipitated with acetonitrile or ethanol and centrifuged (2200*g*, 3 min). Degradation of the ^68^Ga complexes was evaluated by RP-HPLC. Samples from DTPA and FeCl_3_-containing solutions were injected onto the HPLC directly.

### 2.6 In vitro uptake assays

In vitro uptake of ^68^Ga-labelled siderophores was determined both in time and with excess of ferri-siderophore as well as NaN_3_ to block energy-dependent uptake. For the monitoring of uptake in time, ^68^Ga-siderophores were incubated with iron-deficient or iron-sufficient *A.f.* mycelia up to 90 min at RT with or without blocking solution (ferri-siderophore) in 96-well filter plates (Millipore, Massachusetts, USA). Incubation was interrupted by filtration of the medium and rapid rinsing with ice-cold tris(hydroxymethyl)aminomethane (TRIS) buffer. Filters were collected and counted in a γ-counter. For the testing of energy-dependent uptake, ^68^Ga-labelled siderophores were incubated again with iron-deficient or iron-sufficient *A.f.* mycelia for 45 min at RT with and without NaN_3_ or excess of ferri-siderophore in 96-well filter plates. Incubation was interrupted by filtration of the medium and rapid rinsing with ice-cold TRIS buffer. Filters were collected and counted in a γ-counter.

### 2.7 Utilization of siderophores by *Aspergillus fumigatus*

To measure utilization of siderophores by *A.f.* via growth assays, desferri-siderophores were added to 2 ml/well minimal medium agar containing 10 μM FeSO_4_ in 12-well tissue culture plates. Aliquots of 10^4^ conidia of the *A.f.* mutant strain *ΔsidAΔftrA*, the growth of which is supported only in the presence of utilizable siderophores [20], were point inoculated and growth scored after incubation at 37°C for 24 and 48 h, respectively. The same plate without siderophores served as a control.

### 2.8 Biodistribution in normal Balb/c mice

Animal experiments were performed with the permission of the Austrian Ministry of Science (66011) and in accordance with regulations of the Austrian Animal Protection Laws. Biodistribution of ^68^Ga-siderophores was studied in normal (non-infected) Balb/c mice. ^68^Ga-labelled siderophores (∼2 MBq/mouse, corresponding to 0.1–0.2 μg of siderophore) were injected into the tail vein. The first group of mice (*n*=3) was sacrificed by cervical dislocation 30 min pi, followed by the second group of mice (*n*=3) 90 min pi. Different organs and tissues (blood, spleen, pancreas, stomach, intestine, kidneys, liver, heart, lung, muscle, femur) were removed and collected. The amount of radioactivity for each sample was determined using a γ-counter. Obtained data were expressed as a percentage of injected dose per gram of organ (%ID/g).

### 2.9 Statistical analysis

The in vitro uptake data were compared using *t* test (level of significance, *P*<.01). Analysis was performed using the Microsoft Office Excel 2007 program.

## 3 Results

### 3.1 ^68^Ga labelling of selected siderophores

**Fig. 1:**
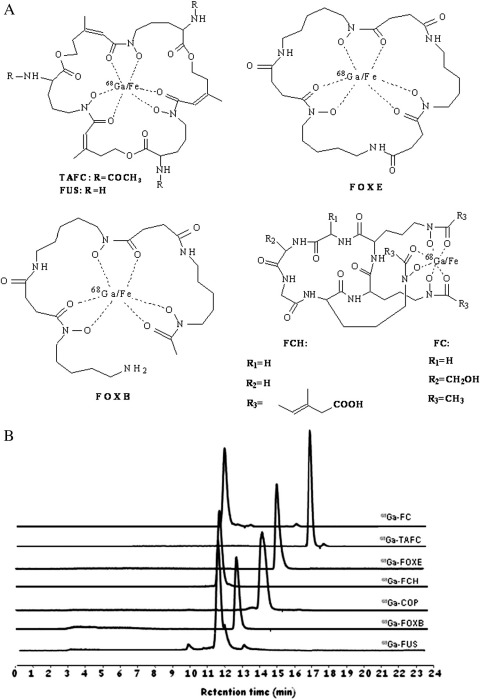
(A) Chemical structures of ^68^Ga/Fe-siderophores. (B) HPLC-Radiochromatograms (RP-C18, ACN/H_2_O/0.1%TFA gradient) of studied ^68^Ga-siderophores.

Certain differences were observed in labelling conditions and efficiency of studied desferri-siderophores. Coprogen (COP), ferrichrome (FCH) and TAFC were labelled with ^68^Ga using sodium acetate as a buffer at RT for less than 15 min with RCP >90%. Ferrioxamine B (FOXB) and ferrioxamine E (FOXE) radiolabelling was performed in sodium acetate at 80°C for 20 min with RCP >90 %, whereas FC and FUS labelled using sodium acetate at RT for 15 min showed lower radiolabelling efficiency at ∼80% (Fig. 1A and B).

### 3.2 Log *P*, protein binding and stability studies

All radiolabelled siderophores showed hydrophilic properties (log *P*=−1.65 to −3.56). Protein binding values up to 120 min of incubation time did not exceed 22% for ^68^Ga-COP, FOXB, FOXE, FUS and TAFC. ^68^Ga-FC and ^68^Ga-FCH showed high protein binding values even after 30 min of incubation. Stability studies revealed the instability of the majority of studied ^68^Ga-siderophores under tested conditions except for ^68^Ga-TAFC and ^68^Ga-FOXE, which showed excellent stability in all examined media (Table 1).

### 3.3 In vitro uptake studies

#### 3.3.1 In vitro uptake studies in time

**Fig. 2:**
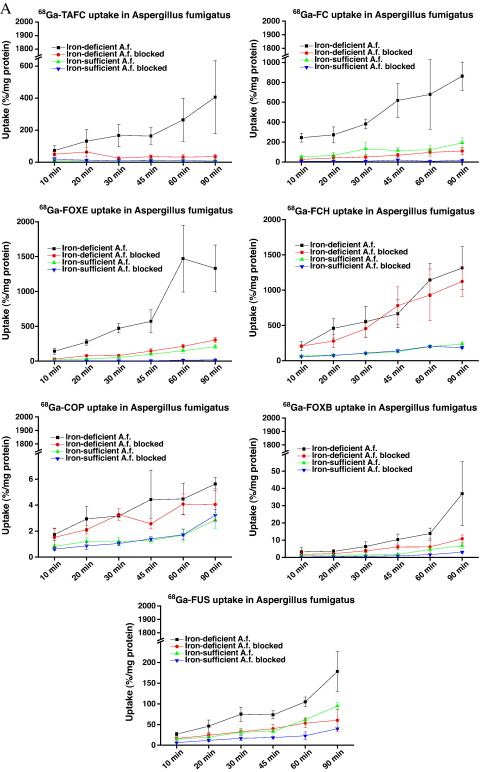

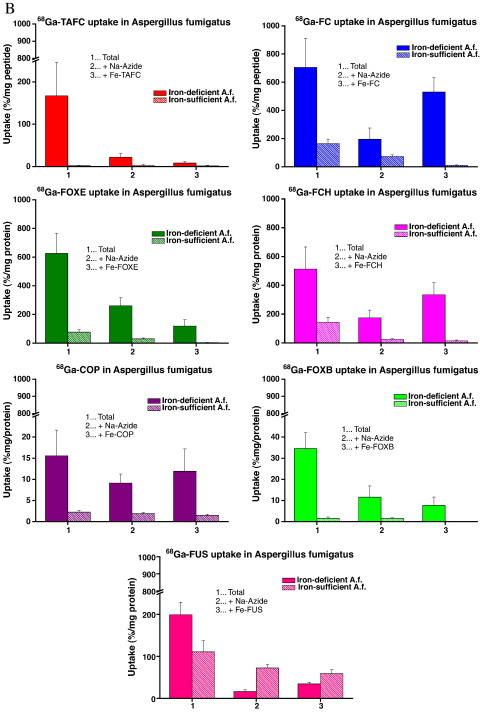
(A) In vitro uptake of ^68^Ga-labelled siderophores in *A.f.* cultures over time (mean±S.D., *n*=4). Incubation in iron-sufficient media as well as addition of excess of ferri-siderophore statistically significantly reduced the uptake (*P*<.01), except for early time points (10–20 min), and ^68^Ga-FCH, ^68^Ga-COP and ^68^Ga-FOXB. (B) In vitro uptake of ^68^Ga-labelled siderophores in the presence of excess of NaN_3_ and ferri-siderophore (mean±S.D., *n*=8). Incubation in iron-sufficient media and addition of sodium azide statistically significantly reduced the uptake (*P*<.01) for all tested ^68^Ga-siderophores.

Uptake of ^68^Ga-siderophores by *A.f.* was highly dependent on the mycelial iron load and type of siderophore. Under iron-deficient conditions ^68^Ga-FC, FCH, FUS, FOXE and TAFC showed rapid uptake increasing over time (up to 90 min) that could be blocked using siderophore ferri-form, while ^68^Ga-COP and ^68^Ga-FOXB displayed negligible uptake in both iron-deficient and iron-sufficient *A.f.* cultures (Fig. 2A).

#### 3.3.2 In vitro studies of uptake energy dependence

Studies of uptake energy dependence confirmed the results of in vitro uptake studies in time. Furthermore, these experiments revealed that uptake of tested ^68^Ga-siderophores in iron-deficient *A.f.* cultures can be blocked with excess of NaN_3_, indicating energy-dependent uptake mechanism (Fig. 2B).

### 3.4 Utilization of siderophores by *Aspergillus fumigatus*

**Fig. 3:**
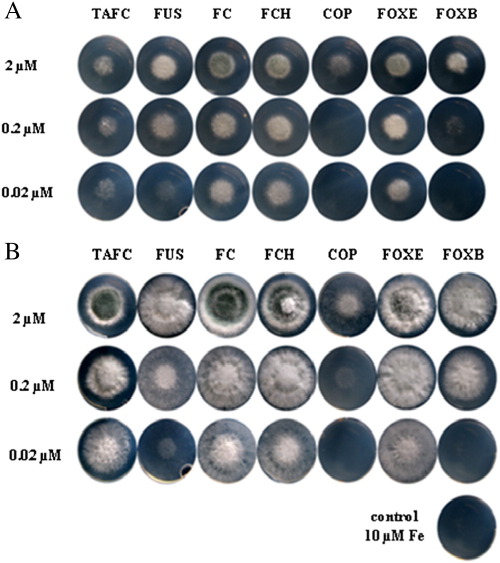
Growth stimulation by siderophores of an *A.f.* mutant strain lacking siderophore biosynthesis and reductive iron assimilation (*ΔsidAΔftrA*). Aliquots of 10^4^ conidia were point-inoculated on minimal medium containing 10 μM iron, and the indicated concentration of siderophores and pictures were taken after incubation for 24 h (A) and 48 h (B) at 37°C growth. The control without siderophore supplementation demonstrates the siderophore-dependent growth phenotype of *ΔsidAΔftrA*. Sporulation is indicated by the green-greyish colouring attributed to the green spore pigment, especially pronounced after 48 h by 2 μM TAFC, FC, FCH and FOXE.

Utilization of iron chelated by different siderophores was studied using the *A.f.* strain *ΔsidAΔftrA* [20]*.* This mutant strain lacks siderophore biosynthesis and reductive iron assimilation, and, therefore, its growth depends on externally supplied siderophore–iron. Growth assays demonstrated that *A.f.* is able to utilize not only the endogenous siderophores TAFC, FUS and FC, but also the xenosiderophores FCH, COP, FOXE and FOXB (Fig. 3). In agreement with the low uptake found in the short-term in vitro uptake assays (Fig. 2A), however, COP and FOXB supported growth after 24 h of incubation in low concentrations (>10–100-fold higher as the other siderophores) (Fig. 3A). Similarly, *A.f.* growth was supported to a lower degree after 48 h of incubation by COP and FOXB and, at this time, FUS was also less efficient as iron source (Fig. 3B). TAFC, FC, FCH and, to a lower degree, FOXE (but not COP, FUS and FOXB) supported *A.f.* sporulation (Fig. 3, best seen at 2 μM siderophore).

### 3.5 Biodistribution in normal Balb/c mice

**Fig. 4:**
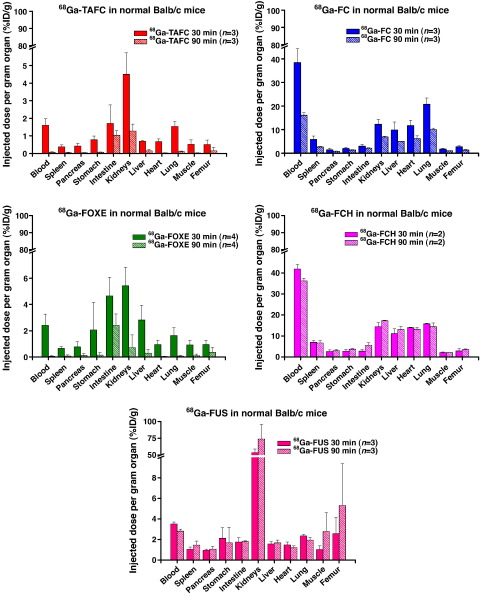
Biodistribution of ^68^Ga-labelled siderophores in normal Balb/c mice.

^68^Ga-TAFC and ^68^Ga-FOXE in mice showed rapid renal excretion, low blood values (1.6±0.37 or 2.4±0.85 %ID/g 30 min) and scarcely any retention in other organs even at a short period (90 min) after application. ^68^Ga-FC and ^68^Ga-FCH displayed significant retention in blood (16.1±1.07 or 36.2±1.25 %ID/g 90 min) and in some major organs. ^68^Ga-FUS revealed relatively low blood levels (3.5±0.21 %ID/g 30 min), but very high retention in kidneys (73.8±21.88 %ID/g 90 min) (Fig. 4).

## 4 Discussion

Invasive fungal and bacterial infections are a major cause of morbidity and mortality in neutropenic patients. In recent years, several cancer centres have reported an increase in the incidence of infections caused by difficult-to-treat opportunistic molds such as *Aspergillus, Candida*, *Zygomycetes*, *Fusarium* and *Scedosporium* species, and yeasts such as *Trichosporon* species [21]. Infections associated with *Aspergillus* spp. are one of the most serious, because of limitations in diagnosis resulting in high mortality. *A.f.* is by far the most important pathogenic *Aspergillus* species known. It is the main *Aspergillus* species responsible for IA.

Transplant recipients, patients under immunosuppressive or steroid therapy, and patients with HIV infection, cystic fibrosis, chronic granulomatous disease and acute leukemia are among the most significant groups of immunocompromised hosts at high risk of IA. The highest risk is in neutropenia, where lungs are affected in 90% of cases [22]. Although a number of diagnostic techniques are presently applied and alternate diagnostic strategies have been investigated even in the radiopharmaceutical field (^99m^Tc-labelled PEG-liposomes, ^99m^Tc-interleukin 8, ^99m^Tc-fluconazole, ^99m^Tc-ubiqucidin or ^111^In-labelled hyphae-binding peptide (c(CGGRLGPFC)-NH_2_)) [18], a sufficiently specific and sensitive tool is currently missing.

PET is a very sensitive technique for noninvasive imaging of molecular processes and is used for a variety of application especially in oncology. The recent interest in the positron emitter ^68^Ga opens new applications in diagnostic imaging with increased sensitivity and specificity [17]. After very promising results of our study with ^68^Ga-TAFC in a rat IA model [18], proving the principle of IA-PET imaging using ^68^Ga-labelled siderophores, we focused on the selection, characterisation and optimization of the most promising candidates for diagnostic applications as a basis for clinical implementation of PET in imaging *A.f.* infections. Here, we reported the in vitro and in vivo evaluation of selected siderophores showing two promising candidates for detection of IA.

FOXE and TAFC could be both labelled with ^68^Ga at high specific activities, although the labelling protocols differ. Both siderophores showed hydrophilic properties with excellent in vitro stability and low values of protein binding. Other siderophores (COP, FC, FCH, FUS, FOXB) included in this study displayed more or less pronounced instability especially in human serum and in the presence of DTPA excess. High values of protein binding as well as the instability in human serum and towards DTPA challenge observed for ^68^Ga-FC and ^68^Ga-FCH are in concordance with high activity levels in blood, which were found in in vivo biodistribution studies. This indicates an in vitro and in vivo transchelation of ^68^Ga to transferrin. Rapid in vitro uptake was observed in *A.f.* iron-deficient cultures for ^68^Ga-FOXE and ^68^Ga-TAFC that could be blocked with excess of siderophore ferri-form and sodium azide. ^68^Ga-FC, ^68^Ga-FCH and ^68^Ga-FUS showed not only high uptake in iron-deficient mycelia, but also certain uptake in iron-sufficient media, which could be only partially blocked using the respective ferri-siderophore, indicating some unspecific binding. Sodium azide addition resulted in reduction of uptake in all cases; however, the extent of reduction varied and, for example, was only about 40% in the case of ^68^Ga-FOXE. This phenomenon is in concordance with data published by Protchenko et al. [23], indicating that in iron-deficient conditions not only transporters are up-regulated, but also siderophore binding proteins, leading to increased cell surface binding of Fe (and ^68^Ga) siderophores. ^68^Ga-COP and ^68^Ga-FOXB revealed low uptake in both iron-deficient and iron-sufficient *A.f.* cultures. In agreement, COP and FOXB displayed low *A.f.* growth stimulation compared to the other siderophores. Therefore COP and FOXB were excluded from the following in vivo studies. Biodistribution behaviour of ^68^Ga-FOXE and ^68^Ga-TAFC in normal mice was comparable with rapid renal excretion and low blood levels 90 min pi. In the case of ^68^Ga-FC and ^68^Ga-FCH, high blood values and activity retention in some organs were observed even 90 min after injection, indicative of in vivo instability and ^68^Ga transchelation to transferrin. This clearly correlates with their protein binding data and makes them unfitting agents for IA imaging. In vivo results of ^68^Ga-FUS displayed very high uptake and retention in kidneys and with its inferior in vitro stability also seems to be unsuitable for *A.f.* infection imaging.

Our data clearly show that from this series of siderophores ^68^Ga-TAFC and ^68^Ga-FOXE are the most promising compounds for *A.f.* infection imaging. However, these data do not allow judgment as to which of the two compounds may be superior. Further studies in this respect are needed and are currently ongoing.

## 5 Conclusion

We have shown in this work that a number of different siderophores bind ^68^Ga with high affinity under mild conditions. Especially ^68^Ga-TAFC and ^68^Ga-FOXE displayed high in vitro stability and convenient in vivo behaviour for their intended application. In combination with their excellent and specific uptake in *A.f.* cultures, they present great potential as radiopharmaceuticals for *Aspergillus* infection imaging. These two promising compounds are currently investigated for imaging sensitivity in animal models of *A.f.* infection, pathogen selectivity and toxicity.

## Figures and Tables

**Table 1:** In vitro characteristics of studied ^68^Ga-siderophores

^68^Ga-Siderophore	Log P (mean±S.D., *n*=6)	Incubation time (min)	Protein binding (%) (mean, *n*=2)	Stability in human serum (%) (*n*=1)	Stability in 0.1 M FeCl_3_ (%) (*n*=1)	Stability in 6 mM DTPA (%) (*n*=1)
^68^Ga-TAFC	−2.59±0.15	30	0.47	99.9	99.4	85.0
		60	0.76	99.9	98.5	84.7
		120	1.21	99.9	99.3	81.8
^68^Ga-FC	−3.17±0.03	30	58.74	53.7	91.3	68.5
		60	55.73	48.4	93.7	57.3
		120	64.36	37.3	92.2	35.3
^68^Ga-FOXE	−1.65±0.03	30	0.27	99.9	92.9	94.3
		60	0.24	99.9	91.8	93.8
		120	0.53	99.9	94.5	93.2
^68^Ga-FCH	−3.24±0.07	30	60.24	85.5	78.9	73.8
		60	57.26	83.8	77.9	52.7
		120	60.88	84.8	76.1	19.9
^68^Ga-COP	−2.77±0.07	30	0.55	99.2	92.5	69.9
		60	0.68	99.3	94.4	65.9
		120	0.82	98.7	94.3	65.5
^68^Ga-FOXB	−3.56±0.17	30	7.67	74.1	51.5	60.1
		60	10.29	72.0	56.4	54.5
		120	10.83	75.4	58.7	52.9
^68^Ga-FUS	−2.73±0.01	30	12.87	71.5	94.3	80.6
		60	16.81	68.0	93.9	79.4
		120	21.48	67.8	94.8	76.9
